# Inflation Lines of Endotracheal Tubes: How Long Should It Be?

**DOI:** 10.5005/jp-journals-10071-23229

**Published:** 2019-08

**Authors:** Summit Dev Bloria, Pallavi Bloria

**Affiliations:** 1 Department of Anesthesia, PGIMER, Chandigarh, India; 2 Department of Anesthesia, Government Medical College, Jammu, Jammu and Kashmir, India

## Abstract

**How to cite this article:** Bloria SD, Bloria P. Inflation Lines of Endotracheal Tubes: How Long Should It Be? Indian J Crit Care Med 2019;23(8):389.

Endotracheal tubes have revolutionized the practise of anesthesia and critical care by providing an effective separation between the airway and the esophagus. Cuffed PVC endotracheal tubes are used to provide anesthesia as well as critical care management of adults and even pediatric patients. However, a known problem with the use of cuffed endotracheal tubes is severing/cutting/damage to the inflation line compromising with ventilation and safety of patient and necessitating change of ETT.

We wish to highlight, what we believe, is an error in designing of standard cuffed endotracheal tubes and is the cause of many airway related complications. We believe a small alteration in the design of ETTs will reduce the incidence of cuff failure and make the practise of anesthesia safer.

The high volume low pressure cuff of endotracheal tube is connected to the pilot balloon and one way valve by means of an inflation line. Although there is some variation between various manufacturers, in most of the cuffed PVC ETTs, the inflation line arises from the wall of PVC ETT roughly at the center of the length of the endotracheal tube.

After taking origin from the ETT, the average length of inflation line till it joins the pilot balloon is approximately 15 cm, some minor variations notwithstanding. We could not find any purpose of such long inflation lines. Even the ASTM criteria specify only 3 cm as the minimum distance between the machine end of the inflation tube and the pilot balloon.

We believe such long hanging inflation lines predispose to failure of cuff inflation mechanism of ETT. In one of our ICU patients, the inflation line was accidently cut by the barber while shaving and we had to change the ETT. Literature is replete with incidences of severed inflation lines for which many methods of tiding over the scenario are suggested like using an IV cannula and a 3-way;^1^ a blunt tipped needle and a 3 way;^2^ epidural filter;^3^ inflation line of an unused ETT ^4^, etc. Although none of the authors perceived the long length of cuff inflation line as the cause of complication, that the longer cuff lines are more likely to get damaged is a simple logical reasoning. Finally, Bhandari et al. do suggest that increased length of inflation tube of the ETT may increase the chances of accidental pulling and disruption of inflation system.^5^

We propose that the inflation lines should arise from the ETT at around the proximal one-fourths of its length (from machine end) and the total length of the inflation lines should not exceed around 5 cm (Fig. 1).

We believe that shifting the origin of inflation lines proximally and shortening its length will decrease the incidence of cuff deflation due to severance of inflation line.

It must be noticed that there already are other types of endotracheal tubes with inflation line exiting more proximally like reinforced tube, RAE tube (nasal). In case of reinforced tubes, the inflation line leaves the ETT body just distal to distal end of connector. We believe that this design should be applied to the standard ETTs also.

**Fig. 1:**
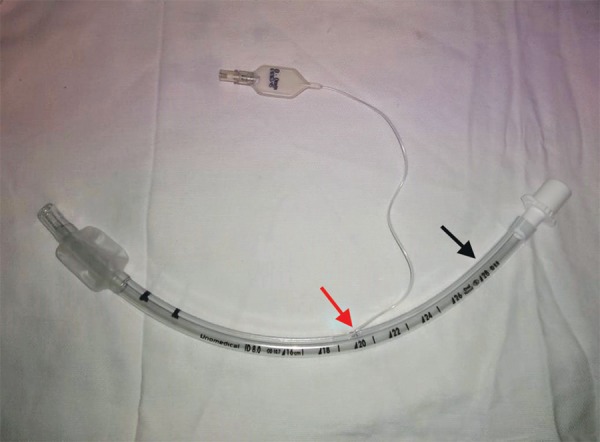
The red arrow denotes the usual site of origin of inflation line. We believe shifting the origin of inflation line more toward the machine end of the ETT will help decrease the chances of failure of inflation system
